# Alcohol and Cannabis Use Disorder Symptom Severity, Conduct Disorder, and Callous-Unemotional Traits and Impairment in Expression Recognition

**DOI:** 10.3389/fpsyt.2021.714189

**Published:** 2021-09-20

**Authors:** Robert James R. Blair, Johannah Bashford-Largo, Ru Zhang, Avantika Mathur, Amanda Schwartz, Jaimie Elowsky, Patrick Tyler, Christopher J. Hammond, Francesca M. Filbey, Matthew Dobbertin, Sahil Bajaj, Karina S. Blair

**Affiliations:** ^1^Center for Neurobehavioral Research, Boys Town National Research Hospital, Boys Town, NE, United States; ^2^Child and Family Translational Research Center, Boys Town National Research Hospital, Boys Town, NE, United States; ^3^Department of Psychiatry, John Hopkins University, Baltimore, MD, United States; ^4^Center for BrainHealth, School of Behavioral and Brain Sciences, University of Texas at Dallas, Dallas, TX, United States

**Keywords:** expression recognition, conduct disorder, callous-unemotional traits, cannabis use disorder, alcohol use disorder

## Abstract

**Background:** Alcohol and cannabis are commonly used by adolescents in the United States. Both alcohol use disorder (AUD) and cannabis use disorder (CUD) have been associated with reduced emotion expression recognition ability. However, this work has primarily occurred in adults and has not considered neuro-cognitive risk factors associated with conduct problems that commonly co-occur with, and precede, substance use. Yet, conduct problems are also associated with reduced emotion expression recognition ability. The current study investigated the extent of negative association between AUD and CUD symptom severity and expression recognition ability *over and above* any association of expression recognition ability with conduct problems [conduct disorder (CD) diagnostic status].

**Methods:** In this study, 152 youths aged 12.5–18 years (56 female; 60 diagnosed with CD) completed a rapid presentation morphed intensity facial expression task to investigate the association between relative severity of AUD/CUD and expression recognition ability.

**Results:** Cannabis use disorder identification test (CUDIT) scores were negatively associated with recognition accuracy for higher intensity (particularly sad and fearful) expressions while CD diagnostic status was independently negatively associated with recognition of sad expressions. Alcohol use disorder identification test (AUDIT) scores were not significantly associated with expression recognition ability.

**Conclusions:** These data indicate that relative severity of CUD and CD diagnostic status are statistically independently associated with reduced expression recognition ability. On the basis of these data, we speculate that increased cannabis use during adolescence may exacerbate a neuro-cognitive risk factor for the emergence of aggression and antisocial behavior.

## Introduction

Substance use is common in the United States with lifetime prevalence rates of alcohol use disorder (AUD) and cannabis use disorder (CUD) of 29 and 6%, respectively (1, 2). Use of alcohol/cannabis during adolescence significantly increases the risk of developing AUD/CUD by adulthood (3). Alcohol use disorder and CUD are both significantly co-morbid with conduct disorder (CD) (4, 5) and associated with a significantly increased risk for aggression (6–9). While the associations between AUD, CUD, CD, and aggression likely at least partly reflects the adverse neurodevelopmental effects of alcohol/cannabis use on the adolescent brain (10, 11), an understanding of the neuro-cognitive underpinnings of these associations remains in its infancy.

A form of neuro-cognitive dysfunction associated with CD and aggression is impaired processing of emotional expressions [for a review of this literature, see (12)]. This manifests as both impaired expression recognition [that may be particularly marked for distress cues (13, 14)] and reduced neural responses, particularly within the amygdala, to distress cues [the sadness and fear of others; for a review of this literature, see (12)]. The suggestion is that the reduced responsiveness to the distress of others should be associated with reduced learning to avoid actions that harm other individuals because the individual finds the “punishment” of the other individual's distress less aversive (12). This results in reduced empathy for the victim, reduced guilt [i.e., the development of what have been termed callous-unemotional (CU) traits; e.g., (15)] and reduced avoidance of actions that harm other individuals [i.e., aggression (16)]. Indeed, the positive relationship between CU traits and aggression has been shown to be mediated by the reduced responsiveness of the amygdala to the distress of other individuals (17).

Impairments in expression processing have also been reported in individuals who have engaged in alcohol and cannabis use (18, 19). Behavioral studies of adults with AUD relatively consistently report impaired expression recognition relative to comparison individuals [for reviews of this literature, see (18, 20, 21)]—though there are conflicting results (22, 23). Neuro-imaging studies, again mostly conducted with adults, have shown that chronic alcohol use is associated with reduced BOLD responses to emotional expressions in prefrontal (e.g., dorsolateral and orbitofrontal cortex), limbic (amygdala, hippocampus, insula, and cingulate cortex), and other regions [e.g., temporal and parietal cortex and striatum (24–27)]. Similarly, behavioral studies in adults with CUD, though less common, also report expression recognition impairment (28–30). Moreover, neuro-imaging work indicates that adolescents and adults with significant cannabis use show reduced medial frontal cortical responses (25, 31) and atypical event-related potentials to emotional facial expressions (32–35).

Notably, though, there are at least three gaps in the AUD/CUD literature. First, very little work has examined expression recognition in adolescents, rather than adults, with AUD/CUD. Yet, face/facial expression processing undergoes considerable neuro-development from adolescence to adulthood (36, 37). Adolescents engaging in AUD/CUD may be particularly compromised by exposure to these compounds. Second, almost no previous work has considered co-use rather than singly addressing the association of cannabis or alcohol use with expression recognition impairments [e.g., (28, 29, 38, 39)]. Co-use of alcohol and cannabis is particularly common in adolescents (40, 41). Previous association of expression recognition impairments in patients with AUD or CUD may represent high use levels of the other substance. Third, and most critically for this study, the majority of the previous work did not assess for comorbid CD (or level of CU traits) despite CD being the most common co-morbidity both in adolescents with CUD and AUD (42–44) and, together with high CU traits, *particularly* associated with compromised expression recognition (16). As such it is possible that previous reports associating AUD/CUD with expression recognition impairment might reflect the biology underpinning antisocial behavior disorders.

In short, the goals of the current study were: (i) To determine, in a sample of adolescents, the extent to which severity of AUD/CUD was associated with the ability to accurately recognize the emotional expressions of other individuals *over and above* any association of CD diagnostic status or CU traits with expression recognition ability; and (ii) To determine, the extent of association between expression recognition ability, AUD/CUD severity, as indexed by scores on the Alcohol Use Disorder Identification Test [AUDIT (45)] and the Cannabis Use Disorder Identification Test [CUDIT (46)], and level of CU traits, as indexed by the Inventory of Callous-Unemotional traits [ICU (47)], was associated with aggression. We predicted that: (i) If AUD and/or CUD is associated with compromised facial expression processing, as indicated by the previous literature [e.g., (20, 28)], then AUDIT and/or CUDIT scores would be inversely related to expression recognition accuracy even in models considering associations with co-morbid CD (and ICU scores); (ii) Expression recognition ability, AUD and CUD severity would be significantly associated with aggression.

## Methods

### Participants

Study participants included 158 youths aged 12.5–18 years from a residential treatment program or the surrounding community. They were recruited as part of a broader study determining neural correlates of youth with behavioral and emotional problems, specifically substance use disorders (at least 40% of the population) and mental health concerns [CD, attention deficit/hyperactivity disorder (ADHD), major depressive disorder (MDD), and generalized anxiety disorder (GAD)]. None of these participants have been included in previous papers on this task. However, a number of the participants have been involved in previously published studies associating specific forms of neuro-cognitive dysfunction with severity of AUD/CUD (25, 48–52). Six youths were excluded due to incomplete data. This resulted in a final sample of 152 youths (107 from the residential treatment program and 45 from the community); *M*_age_ = 16.54 (*SD* = 1.23), *M*_IQ_ = 101.69 (*SD* = 11.65), 56 females. Participants recruited from the residential treatment program were enrolled in a highly supervised residential treatment program where they received random drug testing and did not have access to alcohol or drugs (they were abstinent from any substance for at least 4 weeks prior to testing). See Supplementary Methods for information on recruitment, consent/assent, and exclusion criteria.

### Measures

#### Expression Recognition Task

The facial expression recognition task [Adapted From (53)] featured four basic emotions (angry, happy, sad, and fearful) from the well-validated Pictures of Facial Affect Set (54). Participants viewed static expressions of these four basic emotions. Each face had been morphed with a neutral expression from the same exemplar in 20% increments so that the expressions varied in emotional intensity from 100% (0% neutral) to 0% (100% neutral). Participants saw 240 expressions total (4 expressions 12 exemplars 5 intensity levels) presented in random order (i.e., rather than seeing each face morph from 0 to 100%). Each expression appeared for 200 ms and was followed by a response choice screen that required participants to make a forced choice among four possible responses: angry, happy, sad, and fearful. Participants' responses were self-paced. Following each response, a fixation cross (500 ms) appeared, followed by the next expression.

#### Substance Use Disorder Assessments

Participants completed both the AUDIT (55) and CUDIT (46) assessing symptom levels over the previous 12 months. These scales assess overall symptom severity of AUD and CUD, respectively, including overall quantity/frequency of use, abuse symptoms, and dependence symptoms. They show high validity, as higher scores on these scales are associated with a high likelihood of an AUD and/or CUD diagnosis, respectively (45, 46). Cigarette smoking status was determined via the Monitoring the Future Survey (56). Although participants were subject to random urine drug screening as part of the treatment program, they were not drug tested on the day of testing.

#### Aggression Assessments

Participants completed the Reactive–Proactive Aggression Questionnaire [RPQ (57)] and the Inventory of Callous-Unemotional Traits [ICU (47)]. The RPQ indexes the participant's level of reactive and proactive aggression. The ICU indexes CU traits which are positively associated with an increased risk for reactive but particularly proactive aggression (15).

### Statistical Analyses

#### Clinical Characteristics

Descriptive statistics were calculated for all demographic and clinical variables. Correlations were performed to examine potential associations between AUDIT and CUDIT scores and: (i) demographic variables (age, IQ); (ii) smoking [smoking scores ranged from 0 (“Never”) to 4 (“Regularly now”) based on the Monitoring the Future survey; (58)]; and (iii) aggression (RPQ scores). For sex, diagnostic status (CD, ADHD, MDD, and GAD) and medication prescriptions (stimulants, SSRIs, and anti-psychotics), significance of group differences (males vs. females, and cases vs. not cases) in AUDIT/CUDIT scores were determined by ANOVA.

#### Testing the Associations of AUD, CUD, CD Diagnostic Status, and CU Trait Severity With Expression Recognition Accuracy. Covariate Analysis

This was tested via a 2 (Group CD: CD diagnosis; no CD diagnosis)-by-2(Sex)-by-4 (Emotion: Angry, Happy, Sad, Fearful)-by-5 (Morph Intensity: 0, 20, 40, 60, 80, 100%) ANCOVA on participants' expression accuracy with AUDIT, CUDIT, ICU, and IQ scores and age used as continuous covariates. Given the number of clinical variables and thus constraints on power and the absence of a priori predictions with respect to interactions between the clinical variables, these were not included in the model. Given potential concerns regarding skewness/kurtosis of AUDIT/CUDIT scores, this analysis was repeated using Rankit transformed AUDIT and CUDIT scores.

#### Group-Based Analysis

Due to potential concerns regarding individual difference analyses given test-retest issues on many neuropsychological tasks (59, 60), the analyses were repeated using a group-based approach. Participants were grouped according to whether they met, or did not meet, clinical cutoffs on the AUDIT and/or CUDIT suggestive of adolescent AUD (AUDIT ≥ 4) or CUD [CUDIT ≥ 8 (45, 61)]. As such, the test involved a 2 (Group CD: CD diagnosis; no CD diagnosis)-by-2 (Group AUDIT: AUDIT ≥ 4; AUDIT < 4)-by-2(Group CUDIT: CUDIT ≥ 8; CUDIT < 8) ANOVA. Due to probable small cell sizes for some interactions, no inter-group interactions were included in this model.

#### Testing the Association of Expression Recognition, AUD, CUD, and CU Trait Severity With Aggression

This was tested via a univariate ANCOVA on aggression level as indexed by the RPQ total score with accuracy scores for sad, fearful, happy and angry expressions, AUDIT, CUDIT, and ICU scores as covariates.

#### Potential Confounds: Psychiatric Comorbidities

Given potential relationships between AUDIT/CUDIT scores and diagnostic status for other common psychiatric conditions within this sample (ADHD, MDD, GAD), our main ANCOVA was repeated three times with the addition of a group variable corresponding to diagnostic status for these conditions.

#### Medication Status

Given potential relationships between AUDIT/CUDIT scores and medication status within this sample (stimulants, SSRIs, or anti-psychotic medications), our main ANCOVA was repeated three times with the addition of a group variable corresponding to the prescribed use (or not) of these medications.

All analyses were conducted within SPSS 26.0.

## Results

### Clinical Characteristics

Of the 152 participants, 91 endorsed past-year use of either alcohol and/or cannabis. AUDIT scores ranged from 0 to 22 (*M* = 2.61, *SD* = 4.62) and CUDIT scores from 0 to 32 (*M* = 6.74, *SD* = 8.68). Sixty met the clinical cutoffs on the AUDIT and/or CUDIT suggestive of adolescent AUD (AUDIT ≥ 4) or CUD [CUDIT ≥ 8 (45, 61)]. Fifty-five had a CUDIT score ≥8 and 34 participants had an AUDIT score ≥ 4. Sixty-one participants had a CUDIT score = 0 *and* an AUDIT score = 0. AUDIT, but not CUDIT scores, showed significant skewness and kurtosis (respectively, 2.55 and 6.65 for AUDIT and 1.05 and −0.074 for CUDIT). Rankit transformation of AUDIT and CUDIT scores brought levels of skewness and kurtosis to acceptable levels (respectively, 0.86 and −0.25 for AUDIT and 0.80 and −0.39 for CUDIT).

Correlation analyses revealed a strong positive relationship between AUDIT and CUDIT scores (*r* = 0.597, *p* < 0.001—though variance inflation factors were <1.5; 1.07 and 1.10 for AUDIT and CUDIT, respectively). There were also significant correlations for AUDIT and CUDIT scores and tobacco smoking, IQ, and RPQ scores; see Table 1. Males and females significantly differed in AUDIT scores (females > males). All groups of cases corresponding to psychiatric diagnostic status differed in CUDIT scores (cases > non-cases; albeit *p* = 0.06 for GAD). All groups of cases corresponding to psychiatric diagnostic status differed in AUDIT scores (cases > non-cases; albeit *p* = 0.07 for ADHD). Groups differing according to SSRI medication prescription status significantly differed in CUDIT (*p* = 0.01) and AUDIT scores (*p* = 0.03). Groups differing according to antipsychotic medication prescription status significantly differed in CUDIT scores (*p* = 0.02) (for full details, see Table 1).

**Table 1:** Demographic and clinical variables.

	**Mean**	**SD**	***r*** **with CUDIT**			***r*** **with AUDIT**		
AUDIT	2.61	4.62	0.597^**^			–		
CUDIT	6.74	8.68	–			0.597^**^		
Smoking	1.13	1.44	0.618^**^			0.571^**^		
Age	16.54	1.23	0.063			0.154		
IQ	101.69	11.65	−0.167^*^			−0.211^**^		
RPQ total	9.48	7.19	0.376^**^			0.275^**^		
RPQ instrumental	7.40	4.75	0.283^**^			0.135		
RPQ reactive	2.12	3.09	0.388^**^			0.327^**^		
ICU score	22.52	8.84	0.313^**^			0.171^*^		
			**CUDIT mean scores (SD)**			**AUDIT mean scores (SD)**		
			**Male/Case**	**Fem/Not case**	**F (*p*)**	**Male/Case**	**Female/Not case**	**F (*p*)**
Sex	96 males		7.07 (8.87)	6.16 (8.39)	0.39 (*p* = 0.53)	2.00 (3.70)	3.64 (5.77)	4.58 (*p* = 0.03)
CD	60 cases		10.75 (8.84)	4.12 (7.54)	24.47 (*p* < 0.001)	4.00 (5.25)	1.70 (3.93)	9.54 (*p* = 0.002)
ADHD	74 cases		9.46 (8.98)	4.15 (7.58)	15.55 (*p* < 0.001)	3.30 (5.40)	1.95 (3.66)	3.28 (*p* = 0.07)
MDD	20 cases		10.45 (1.35)	6.17 (8.30)	4.30 (*p* = 0.04)	4.85 (7.15)	2.27 (4.04)	5.60 (*p* = 0.02)
GAD	40 cases		8.95 (8.69)	5.95 (8.58)	3.59 (*p* = 0.06)	3.87 (5.66)	2.15 (4.13)	4.1 (*p* = 0.04)
SAD	35 cases		9.86 (9.13)	5.80 (8.36)	6.07 (*p* = 0.02)	5.37 (6.40)	1.78 (3.58)	18.14 (*p* < 0.001)
PTSD	16 cases		7.56 (8.54)	6.64 (8.73)	0.16 (*p* = 0.69)	4.06 (5.22)	2.43 (4.54)	1.79 (*p* = 0.18)
Cocaine/Crack	9 cases		19.11 (6.86)	5.96 (8.20)	22.15 (*p* < 0.001)	6.89 (6.95)	2.34 (4.33)	8.64 (*p* = 0.007)
Meth	6 cases		21.50 (6.86)	6.13 (8.22)	2.38 (*p* < 0.001)	8.83 (6.21)	2.01 (3.77)	24.83 (*p* < 0.001)
Inhalants	4		13.50 (7.00)	6.55 (8.67)	2.52 (*p* = 012)	5 (6.00)	2.54 (4.59)	1.10 (*p* = 0.30)
Stimulants	17 prescribed		9.06 (8.92)	6.44 (8.64)	1.37 (*p* = 0.24)	2.76 (5.29)	2.59 (4.55)	0.02 (*p* = 0.88)
SSRIs	22 prescribed		11.23 (9.35)	5.98 (8.37)	7.16 (*p* = 0.01)	4.59 (5.84)	2.27 (4.32)	4.87 (*p* = 0.03)
Anti-psychotics	9 prescribed		10.22 (9.34)	6.52 (8.63)	1.55 (*p* = 0.02)	2.44 (3.09)	2.62 (4.71)	0.01 (*p* = 0.92)

*SD, standard deviation (also in brackets); AUDIT, alcohol use disorder identification test; CUDIT, cannabis use disorder identification test; RPQ, reactive–proactive aggression questionnaire; CD, conduct disorder; ADHD, attention deficit hyperactivity disorder; MDD, major depressive disorder; GAD, generalized anxiety disorder; Male/Case, column containing data on males or participants who were cases with respect to that psychiatric or medication status; Fem/Not case, column containing data on females or participants who were not cases with respect to that psychiatric or medication status; F, F-value; p, p-value (F and ps correspond to significance of group differences with respect to sex, CD diagnostic status, ADHD diagnostic status, etc.).*

* *significant at p < 0.05,*

** *significant at p < 0.001*.

### Testing the Associations of AUD, CUD, and CU Trait Severity and CD Diagnostic Status With Expression Recognition Accuracy

With respect to the primary aims of the current study, both the Morph Intensity-by-CUDIT [*F*_(4, 544)_ = 2.72, *p* = 0.03, η^2^ = 0.020] and Morph Intensity-by-Emotion-by-CUDIT interactions were significant [*F*_(12, 1, 632)_ = 2.60, *p* = 0.002, η^2^ = 0.019]. Increasing CUDIT scores were significantly negatively associated with recognition of higher intensity (particularly sad and fearful expression) morphs: *r*_Sad:20%_ = −0.19, *r*_Sad:40%_ = −0.27, *r*_Sad:80%_ = −0.21, *r*_Fearful:80%_ = −0.19, and *r*_Fearful:100%_ = −0.18. Increasing CUDIT scores were significantly positively associated with recognition of lower intensity angry morphs (*r*_Angry:20%_ = 0.20). AUDIT scores showed no significant interactions with Morph Intensity [*F*_(4, 580)_ = 1.54, *p* = 0.19, η^2^ = 0.011], Emotion [*F*_(3, 435)_ = 1.26, *p* = 0.29, η^2^ = 0.009], or Morph Intensity-by-Emotion [*F*_(12, 1, 740)_ = 0.70, *p* = 0.754, η^2^ = 0.005].

There was also a significant Expression-by-CD interaction [*F*_(3, 408)_ = 3.18, *p* = 0.02, η^2^ = 0.023]; participants with CD were significantly less accurate than those without CD for sad expressions [*t*_(149)_ = 2.29, *p* = 0.02] but not angry, happy, or fearful expressions [*t*_(149)_ = −1.39 to 1.22, *p* = 0.167–0.227]. In addition, there was a significant Morph Intensity-by-ICU interaction [*F*_(4, 544)_ = 2.39, *p* = 0.05, η^2^ = 0.017]; while there was no significant association between expression recognition and ICU score for the 20 and 40% morphs (*r* = −0.077 and −0.029, respectively), there was a significant association between expression recognition and ICU score for the 60, 80, and 100% morphs (*r* = −0.19, −0.21, and −0.25, respectively).

Additional significant findings included main effects of both IQ [*F*_(1, 136)_ = 8.59, *p* = 0.004, η^2^ = 0.059] and sex [*F*_(1, 136)_ = 5.63, *p* = 0.02, η^2^ = 0.040]. IQ was positively associated with expression accuracy (*r* = 0.26) while females were more accurate than males (*M*_female_ = 7.19); *M*_male_ = 6.67). There was also a significant Morph Intensity-by-Sex interaction [*F*_(4, 544)_ = 3.62, *p* = 0.006, η^2^ = 0.026]; males were less accurate than females for all morph intensities except 20%.

The results of the repetition of this analysis, involving rankit transformed CUDIT and AUDIT scores, mirrored the results of the analysis reported above (see Supplementary Material).

### Group-Based Analysis

The results of the group-based ANOVA largely confirmed those of the ANCOVA outlined above (for details on the demographics of these groups see Supplementary Table 1). There was a significant CUDIT Group-by-Intensity interaction [*F*_(4, 588)_ = 2.85, *p* = 0.02, η^2^ = 0.019] though the CUDIT Group-by-Morph Intensity-by-Emotion-interaction was not significant [*F*_(12, 1764)_ = 1.54, *p* = 0.10, η^2^ = 0.010]. The Expression-by-CD interaction was also significant [*F*_(3, 441)_ = 3.86, *p* = 0.01, η^2^ = 0.026]. There were no significant main effects or interactions with AUDIT Group.

### Testing the Association of Expression Recognition, AUD, CUD, and CU Trait Severity With Aggression

Pearson correlation analyses revealed significant positive associations between aggression as indexed by the RPQ and both AUDIT and CUDIT scores (see Table 1) as well as ICU score (*r* = 0.53; *p* < 0.001) and sadness expression recognition (*r* = −0.17; *p* = 0.041) [though not with recognition of the other expressions; *r* = 0.001, 0.016, and −0.091; *p* = 0.99, 0.085, and 0.276 for angry, happy, and fearful expressions, respectively]. However, our univariate ANCOVA revealed only highly significant associations between ICU and RPQ scores [*F*_(1, 128)_ = 38.67, *p* < 0.001, η^2^ = 0.232] and CUDIT and RPQ scores [*F*_(1, 128)_ = 5.02, *p* = 0.03, η^2^ = 0.038]. There was also an ICU-by-CUDIT score interaction scores [*F*_(1, 128)_ = 5.02, *p* = 0.03, η^2^ = 0.038]. AUDIT was not associated with RPQ scores in our ANCOVA [*F*_(1, 128)_ = 0.69, *p* = 0.41, η^2^ = 0.005] nor was recognition for any of the expressions [*F*_(1, 128)_ = 0.005–0.787, *p* = 0.38–0.94, η^2^ = 0.0–0.006). ICU-by-CUDIT interaction effects were observed, whereby CUDIT scores were positively associated with aggression in individuals whose ICU scores were <29 (*r* = 0.484, *p* < 0.001) but unrelated to aggression in individuals whose ICU scores were 28 or greater (*r* = 0.074, *p* = 0.686). Similarly, ICU scores were positively associated with aggression in individuals whose CUDIT scores were <8 (*r* = 0.592, *p* < 0.001) but significantly less (Steiger's z = 3.47, *p* = 0.0005) associated with aggression in individuals whose CUDIT scores were eight or greater (*r* = 0.315, *p* = 0.023).

#### Potential Confounds: Psychiatric Comorbidities

Our additional ANCOVAs including the addition of a group variable corresponding to diagnostic status ADHD, MDD, GAD, SAD, and PTSD largely replicated our main analysis. In almost all cases, the Morph Intensity-by-CUDIT, Morph Intensity-by-Emotion-by-CUDIT, Expression-by-CD interaction, and Morph Intensity-by-ICU interactions were significant (the exception was the ANCOVA) including ADHD diagnostic status where the Morph Intensity-by-CUDIT and Morph Intensity-by-ICU interactions only reached trend level significance (*p* = 0.057 and 0.068, respectively; for full results, see Supplementary Table 2).

#### Medication Status

Our additional ANCOVAs including the addition of group variables corresponding to the prescribed use (or not) of anti-psychotic, SSRI, or stimulant medications largely replicated our main analysis. In almost all cases, the Morph Intensity-by-CUDIT, Morph Intensity-by-Emotion-by-CUDIT, Expression-by-CD interaction, and Morph Intensity-by-ICU interactions were significant (the exception was the ANCOVA) including SSRI medication status where the Morph Intensity-by-ICU interactions only reached trend level significance (*p* = 0.067; for full results, see Supplementary Table 2).

#### Additional Substance Use

Our additional ANCOVAs including the addition of group variables corresponding to the use (or not) of cocaine/crack, methamphetamine, or inhalants largely replicated our main analysis. In almost all cases, the Morph Intensity-by-CUDIT, Morph Intensity-by-Emotion-by-CUDIT, Expression-by-CD interaction, and Morph Intensity-by-ICU interactions were significant (the exception was the ANCOVA) including cocaine/crack use where the Expression-by-CD interaction only reached trend level significance (*p* = 0.075; for full results, see Supplementary Table 2).

## Discussion

The current study aimed to determine the extent to which severity of AUD/CUD was associated with emotion expressions recognition ability and aggression. This study revealed that: (i) CUDIT (but not AUDIT) scores were associated with reduced expression recognition ability, particularly for sad and fearful facial expressions, over and above reduced expression recognition ability associated with both CD diagnostic status and CU traits and (ii) CUDIT and ICU scores were particularly associated with aggression in this sample.

Reduced expression recognition ability, particularly the ability to recognize distress cues, has long been associated with increased aggression [e.g., (62)] and the presence of CU traits [for a review of this literature, see (12)]. The suggestion is that the reduced response to the distress of others is associated with reduced avoidance of actions that harm other individuals, reduced guilt, and reduced empathy for potential victims (12). The current study, consistent with considerable previous work [for a review, see (12)], revealed that both CD diagnostic status and higher levels of CU traits were associated with reduced expression recognition ability. Within this study, CD diagnostic status was particularly associated with reduced sadness expression ability. This is consistent with fMRI work indicating particularly compromised neural responsiveness to sad facial expressions (63). Level of CU traits was associated with generally reduced expression recognition ability across facial expressions, particularly for “easier” (higher intensity) morphs. While some data have associated CU traits with reduced responsiveness particularly to distress cues [see for a review, (12)], other data has indicated that closely related psychopathic traits are associated with more general reduced expression recognition ability [for a meta-analysis of the literature, see (13)].

The main goal of this study was to determine the extent to which severity of AUD/CUD was associated with emotion expression recognition ability. In particular, our primary aim was to address three gaps in the existing literature on AUD/CUD and expression recognition ability, specifically (i) the relative absence of work examining expression recognition in adolescents, rather than adults, with AUD/CUD; (ii) the relative specificity vs. generality of expression recognition alterations in adolescents in relation to AUD compared to CUD severity; and (iii) the extent to which any associations between AUD/CUD and expression recognition ability existed when potential confounds of CD diagnostic status and level of CU traits were accounted for. As such, the current study revealed that the association between AUD/CUD and reduced expression recognition ability reported in adults [e.g., (18, 19)] is also observed in adolescents. Moreover, it suggested that the association was particularly strong for CUD, relative to AUD, severity, and that this association is present even when potential confounds associated with comorbid CD and co-existing levels of CU traits are taken into account.

We did not specifically predict that there would be a stronger association between CUDIT, relative to AUDIT, scores, and expression recognition ability. Previous work with adults with alcohol use difficulties has relatively consistently associated AUD with reduced expression recognition ability [for reviews of this literature, see (18, 20, 21)]—though there are conflicting results (22, 23). Moreover previous neuro-imaging work, mostly conducted with adults, has shown chronic alcohol use is associated with reduced BOLD responses to emotional expressions in cortical and subcortical regions previously shown to be responsive to emotional expressions (24–27). It is possible that any association between AUD severity and reduced expression recognition ability is notably weaker in adolescents and that only the prolonged, severe exposure seen in adults with AUD-related concerns is associated with reduced expression recognition ability. However, it is also possible that the effects for CUDIT scores but not AUDIT scores reflected a statistical artifact driven by the greater variance in CUDIT scores in this sample (sd_CUDIT_ = 8.68; sd_AUDIT_ = 4.62)—or the restricted variance in the AUDIT scores may have limited the capacity to identify AUDIT-expression recognition associations [though note that this has not been the case in other work investigating other functions in an overlapping sample (48, 51, 64)]. While the results were similar in the group-based ANOVA, there were rather more participants meeting the CUDIT cut-off (*N* = 55) than the AUDIT cut-offs (*N* = 34) and very few participants only meeting the AUDIT but not CUDIT cut-off (*N* = 5). Moreover, and in line with previous work with adolecents (65, 66), co-use of alcohol and cannabis was very common in this sample and the correlation of AUDIT and CUDIT scores was highly significant (even if the variance inflation factors were not high enough to indicate collinearity was a significant concern). As such, it is probably important to be cautious about any definitive conclusions regarding AUD severity and expression recognition ability in adolescents. However, irrespective of conclusions with respect to AUD severity in adolescents, the current results strongly indicate that CUD severity, as indexed by CUDIT score, is associated with reduced expression recognition ability, particularly for distress cues (sad and fearful expressions), and that this reduced ability cannot be accounted for by co-morbid CD diagnostic status or severity of CU traits.

Developmentally, early conduct problems are one of the main predictors of later emergence of substance use disorders (67–69). Reduced response control and atypical reinforcement-based decision-making, seen in individuals with conduct problems [e.g., (70, 71)], have been identified as risk factors for the emergence of substance use disorders (72–74). CU traits, which are particularly associated with reduced expression recognition ability [see for a review (12)], have also been identified as a risk factor for the emergence of substance use disorders (75). The current data indicate that reduced expression recognition may also result from substance (cannabis) use. This is consistent with fMRI data indicating disrupted face processing and reduced emotional responding as a function of CUDIT score in adolescents (25, 52, 76). As such, premorbid/pre-drug exposure neurobehavioral phenotypes associated with CD that convey risk for the development of substance use disorders may potentially be *exacerbated* by cannabis or alcohol exposure during adolescence [given other data indicating that substance negatively impacts the function of regions implicated in response control and reinforcement-based decision-making in adolescents (49, 51)]. In short, substance use may exacerbate adolescent CD. This suggests that particular care may be necessary when treating adolescents presenting with both CD and substance use.

Notably, our ANCOVA analysis on the RPQ data revealed that history of aggression (RPQ score) was associated with ICU and CUDIT scores (though not AUDIT scores). CU traits have long been associated with an increased risk for aggression (77, 78) as has substance use (43, 44). Again, it is necessary to be cautious regarding the contributions of CUD severity relative to AUD severity (given the greater variance in CUDIT scores in this sample; see above). Indeed, in other work we have seen a relationship between AUD severity and disruption in neural systems involved in reactive aggression/retaliation (79). Interestingly, our current findings showed that CU traits and CUD severity have statisically seperable associations with aggression. Furthermore, they identified an interaction in the association of ICU and CUDIT scores with aggression, with ICU scores being robustly correlated with aggression among youth below the clinical cut-off CUDIT score and CUDIT scores being robustly correlated with aggression among youth with levels of self-reported ICU below the cut-off (Frick, personal communication). These data suggest relatively independent pathways to aggressive responding occurring through CU traits and separately through youth cannabis use that become difficult to untangle in individuals who are clinically more severe in either their CU traits or CUD symptom severity. Unfortunately, the absence of a longitudinal design means that we cannot disentangle the chronology of these associations [see above (6)].

The results of this study should be viewed in light of several limitations. First, we did not conduct urine or breathalyzer testing for alcohol or cannabis use on the day of testing. However, this concern is mitigated by the fact that all the participants with significant substance use history were residents of a highly supervised residential treatment facility and subject to random drug testing as part of treatment for at least 4 weeks prior to testing. Second, this study was cross-sectional. As such, the associations reported in the present study might reflect neurotoxic/neuroplastic effects of cannabis use on the developing brain and/or pre-existing risk factors for CUD. Third, there was a high degree of psychiatric co-morbidity in the residential treatment sample and thus the current findings might reflect the psychiatric co-morbidities. Some previous work has excluded participants with psychiatric conditions [e.g., (80–82)]. The problem with this approach is that approximately 80% of adolescents with a SUD present with one or more co-morbid psychiatric conditions (83, 84). Indeed, AUD and CUD are associated with a number of co-morbid psychiatric conditions (85, 86). As such, studies that exclude youth with psychiatric comorbidities are clinically atypical and may not generalize. Moreover, controlling for ADHD, MDD, and/or GAD diagnostic status in our analyses did not significantly alter our main study findings (see Supplementary Table 2). As such, it is unlikely current findings might reflect the psychiatric co-morbidities. Fourth, a number of the study participants were medicated and as such the current findings might reflect this medication status rather than CUD. However, controlling for anti-psychotic, SSRI, or stimulant medication status in our analyses did not significantly alter our main study findings (see Supplementary Table 2). As such, it is unlikely current findings reflect medication usage. Fifth, the AUDIT scores within this sample showed concerning levels of skewness and kurtosis potentially affecting the interpretation of the main ANCOVA analysis. Importantly though, the results of both the dimensional analysis using transformed AUDIT and CUDIT scores and the group-based analysis mirrored the results of our main ANCOVA analysis indicating that the current results are not an artifact of skewness within the AUDIT scores.

In summary, in an adolescent sample with variable levels of psychopathology and substance use, CUDIT scores, CD diagnostic status, and ICU scores were all associated with expression recognition accuracy. Higher CUDIT scores were associated with relatively weaker recognition accuracy for higher intensity (particularly sad and fearful) morphs. Higher ICU scores were also associated with relatively weaker recognition accuracy for higher intensity morphs. CD diagnostic status was associated with poorer recognition of sad expressions. CUDIT scores and ICU scores were also particularly associated with aggression (though the statistical model examining simultaneously associations with aggression, indicated only CUDIT and ICU scores and not expression recognition ability independently of these). The current data are consistent with previous data indicating that CD diagnostic status and ICU scores are associated with reduced expression recognition ability. This reduced ability is thought to underpin the empathy impairments associated with these conditions and increase the risk for aggression. The current data indicate that CUD severity, as indexed by the CUDIT, is also associated with reduced expression recognition ability. As such increased cannabis use during adolescence may exacerbate a neuro-cognitive risk factor for the emergence of aggression and antisocial behavior.

## Data Availability Statement

The raw data supporting the conclusions of this article will be made available by the authors, without undue reservation.

## Ethics Statement

The studies involving human participants were reviewed and approved by Boys Town Institutional Review Board. Written informed consent to participate in this study was provided by the participants' legal guardian/next of kin.

## Author Contributions

All authors contributed to writing the manuscript. JB-L and MD were particularly involved in clinical care of the participants. AS and JE were particularly involved in data collection.

## Funding

This work was supported by Boys Town National Research Hospital. RB was supported by the National Institutes of Health (P20GM130461) and the Rural Drug Addiction Research Center at the University of Nebraska-Lincoln. CH was supported by K12-DA000357 and R34-DA050292. The funders had no role in study design, data collection, data analysis, decision to publish, or manuscript preparation.

## Conflict of Interest

CH receives grant support from the National Institute on Drug Abuse, the American Academy of Child and Adolescent Psychiatry [AACAP Physician Scientist Career Development Award, K12DA000357], the National Network of Depression Centers, and the Armstrong Institute at Johns Hopkins Bayview and serves as a scientific advisor for the National Courts and Science Institute and as a subject matter expert for the Substance Abuse Mental Health Services Administration (SAMHSA). The remaining authors declare that the research was conducted in the absence of any commercial or financial relationships that could be construed as a potential conflict of interest.

## Publisher's Note

All claims expressed in this article are solely those of the authors and do not necessarily represent those of their affiliated organizations, or those of the publisher, the editors and the reviewers. Any product that may be evaluated in this article, or claim that may be made by its manufacturer, is not guaranteed or endorsed by the publisher.
