# Supplementary material for: Alcohol and Cannabis Use Disorder Symptom Severity, Conduct Disorder, and Callous-Unemotional Traits and Impairment in Expression Recognition
Source: Front Psychiatry. 2021 Sep 20;12:714189. doi: 10.3389/fpsyt.2021.714189 (PMC8488132; doi:10.3389/fpsyt.2021.714189)
Supplement: Supplementary file 1 [file Data_Sheet_1.docx]

**Supplemental Materials**

***Supplemental Methods***

***Information on recruitment, consent/assent, and exclusion criteria:*** Youths recruited from Boys Town had been referred for behavioral and mental health problems, including substance use disorders. Clinical assessment occurred shortly after their arrival at Boys Town. Participants from the community were recruited through flyers or social media. Clinical characterization for all participants was done through psychiatric interviews by licensed and board-certified child and adolescent psychiatrists with the participants and their parents/caregivers, to adhere closely to common clinical practice.

The Boys Town National Research Hospital Institutional Review Board approved this study. A doctoral level researcher or a member of the clinical research team obtained written informed consent and assent. In all cases, youth had the right to decline participation at any time before or during the study.

Exclusion criteria for the broader project included IQ<75 assessed with the Wechsler Abbreviated Scale of Intelligence (WASI two-subtest form; Wechsler, 2011), pregnancy, non-psychiatric medical conditions that require the use of medication that may have psychotropic effects (e.g., beta blockers or steroids), current psychosis, pervasive developmental disorders, Tourette’s disorder and neurological disorders. Current psychiatric conditions (other than psychotic disorders or pervasive developmental disorders) were not exclusionary. Use of psychotropic medications for psychiatric indications (e.g., stimulants, selective serotonin reuptake inhibitors) were not exclusory. Note also that being a ward of state was exclusory for participation in this study.

***Supplemental Results***

***Results of the ANCOVA analysis using rankit transformed, rather than raw, CUDIT and AUDIT scores:*** With respect to the primary aims of the current study, both the Morph Intensity-by-CUDIT (F(4, 548) = 2.89, *p* = .02, η^2^ = .021) and Morph Intensity-by-Emotion-by-CUDIT interactions remained significant (F(12, 1644) = 2.88, *p* = .001, η^2^ = .021). The Expression-by-CD interaction also remained significant (F(3, 411) = 3.24, *p* = .02, η^2^ = .023). There interactions of AUDIT scores with Morph Intensity (F(4, 548) = 0.81, *p* = .52, η^2^ = .006), Emotion (F(3, 411) = 1.81, *p* = .14, η^2^ = .013) or Morph Intensity-by-Emotion (F(12, 1644) = 0.79, *p* = .66, η^2^ = .006) remained non-significant.

Supplemental Table S1: Demographic and Clinical Variables of groups made up of participants above/below suggested cut-offs for the AUDIT and CUDIT in adolescents

|  | N | Age | s.d. | IQ | s.d. | N (male) |
| --- | --- | --- | --- | --- | --- | --- |
| CUDIT<8 | 97 | 16.50 | 1.28 | 102.72 | 12.09 | 59 |
| CUDIT≥8 | 55 | 16.60 | 1.14 | 99.87 | 10.70 | 37 |
| AUDIT<4 | 118 | 16.40 | 1.23 | 102.61 | 11.89 | 76 |
| AUDIT≥4 | 34 | 17.02 | 1.10 | 98.50 | 10.30 | 20 |
| CUDIT<8 & AUDIT<4 | 92 | 16.42 | 1.25 | 102.61 | 12.22 | 56 |
| CUDIT<8 & AUDIT≥4 | 5 | 18.07 | 0.59 | 104.80 | 10.16 | 3 |
| CUDIT≥8 & AUDIT<4 | 26 | 16.33 | 1.17 | 102.62 | 10.87 | 20 |
| CUDIT≥8 & AUDIT≥4 | 29 | 16.84 | 1.07 | 97.41 | 10.10 | 17 |

Supplemental Table S2: Results of the follow-up ANCOVA analyses examining the robustness of the main ANCOVA analysis following the addition of individual psychiatric diagnoses (or ADHD & MDD & GAD combined) or the addition of individual prescription status (or anti-psychotic & SSRI & stimulant medication combined).

|  | ADHD | | MDD | | GAD | | SAD | | PTSD | | | | | |  |
| --- | --- | --- | --- | --- | --- | --- | --- | --- | --- | --- | --- | --- | --- | --- | --- |
|  | F = | *p* = | F = | *p* = | F = | *p* = | F = | *p* = | F = | *p* = | | | |  |  |
| Morph Intensity-by-CUDIT | 2.31 | 0.06 | 2.87 | 0.02 | 2.60 | 0.04 | 2.70 | 0.03 | 2.61 | 0.04 | | | |  |  |
| Morph Intensity-by-Emotion-by-CUDIT | 2.49 | 0.00 | 2.67 | 0.00 | 2.66 | 0.00 | 2.63 | 0.00 | 2.48 | 0.00 | | | |  |  |
| Expression-by-CD interaction | 4.04 | 0.01 | 3.26 | 0.02 | 3.31 | 0.02 | 2.81 | 0.04 | 2.67 | 0.05 | | | |  |  |
| Morph Intensity-by-ICU | 2.20 | 0.07 | 2.85 | 0.02 | 2.49 | 0.04 | 2.83 | 0.02 | 3.41 | 0.01 | | | |  |  |
|  | Stimulant | | SSRI | | Anti-psychotic | | Stimulant & SSRI & Antipsychotic | |  |  | | | |  |  |
|  | F = | *p* = | F = | *p* = | F = | *p* = | F = | *p* = |  |  | | | |  |  |
| Morph Intensity-by-CUDIT | 2.51 | 0.04 | 3.22 | 0.01 | 2.64 | 0.03 | 3.07 | 0.02 |  |  | | | |  |  |
| Morph Intensity-by-Emotion-by-CUDIT | 2.56 | 0.00 | 2.83 | 0.00 | 2.67 | 0.00 | 2.82 | 0.00 |  |  | | | |  |  |
| Expression-by-CD interaction | 3.19 | 0.02 | 3.04 | 0.03 | 3.31 | 0.02 | 3.01 | 0.03 |  |  | | | |  |  |
| Morph Intensity-by-ICU | 2.48 | 0.04 | 2.21 | 0.07 | 2.55 | 0.04 | 2.40 | 0.05 |  |  | | | |  |  |
|  | Cocaine/crack | | Methamphetamine | | Inhalants | |  | |  | | | | | |  |
|  | F = | *p* = | F = | *p* = | F = | *p* = |  |  |  |  | | | |  |  |
| Morph Intensity-by-CUDIT | 2.66 | 0.03 | 2.61 | 0.04 | 2.75 | 0.03 |  |  |  |  | | | |  |  |
| Morph Intensity-by-Emotion-by-CUDIT | 2.07 | 0.02 | 2.43 | 0.00 | 2.52 | 0.00 |  |  |  |  | | | |  |  |
| Expression-by-CD interaction | 2.32 | 0.08 | 2.71 | 0.05 | 2.64 | 0.05 |  |  |  |  | | | |  |  |
| Morph Intensity-by-ICU | 2.43 | 0.05 | 2.89 | 0.02 | 2.87 | 0.02 |  | | | |  |  |  | | |
